# Monthly Activity of Phlebotominae Sand Flies in Sistan-Baluchistan Province, Southeast Iran

**DOI:** 10.1673/031.013.15301

**Published:** 2013-12-17

**Authors:** H. Kassiri, E. Javadian, M. Sharififard

**Affiliations:** 1 Department of Medical Entomology and Vector Control, School of Health, Ahwaz Jundishapur University of Medical Sciences, Ahwaz, Iran; 2 Department of Medical Entomology and Vector Control, School of Public Health, Tehran University of Medical Sciences, Tehran, Iran

**Keywords:** ecology, leishmaniasis, Phlebotomus, Sergentomyia

## Abstract

The monthly activity of sand flies, which are vectors of leishmaniasis, was studied from May to October 1997 in three regions (plains, mountainous, coastal) of the Sistan-Bluchistan Province using sticky paper traps. In each village, three houses were selected. 30 sticky traps were installed indoors (bedroom, guestroom, toilet, bathroom) and 30 were installed outdoors (rodent burrows, wall cracks). In total, 8,558 and 1,596 sand fly specimens were collected and identified from outdoors and indoors, respectively. Ten species of *Phlebotomus* and eight species of *Sergentomyia* were collected outdoors, and nine species of *Phlebotomus* and 10 species of *Sergentomyia* were collected indoors. *Phlebotomus papatasi* (Scopoli) (Diptera: Psychodidae) was the predominant species found indoors in the plains region (58.4% of insects caught in the region) and was active during the whole study period. The *P. papatasi* peaks of activity were in early May and early October. *Sergentomyia clydei* (Sinton) was found to be the most abundant species outdoors in the plains region and comprised 64.7% of the total insects caught in the region. *Sergentomyia clydei* and *S. tiberiadis* (Alder, Theodor, and Lourie) were the predominant indoor and outdoor, respectively, species from the mountainous region, making up 19.8% and 35%, respectively, of all the insects caught in the region. *Phlebotomus sergenti* Parrot is a proven vector of urban cutaneous leishmaniasis, and *P. alexandri* (Sinton) is a probable vector of Kala-Azar, and both were collected during this study. *Phlebotomus papatasi* was the most predominant species collected indoors in the coastal region (50.8%), its peak activity was in May. *Sergentomyia sintoni* Pringle was the most predominant species collected outdoors in the coastal region (36.4%), and its peak activity was in October. Awareness of the peak activity times of sand flies can be useful in developing strategies to control the flies.

## Introduction

Leishmaniasis is a zoonotic disease caused by *Leishmania* Ross (Kinetoplastida: Trypanosomatida) and transmitted by Phlebotominae sand flies. Different clinical manifestations of this disease occur as cutaneous leishmaniasis, visceral leishmanisis (also known as Kala-azar), and mucocutaneous leishmaniasis (also known as Espundia). Currently, 88 countries are affected by the disease (Ullah et al. 2009). Iran is an endemic area of leishmaniasis, and the disease is prevalent in 15 provinces (MHME 2007). Recently, leishmaniasis has been reported from large rural and urban areas, showing wide diversity and the adaptation of parasites *(Leishmania)* and vectors to the climatic and environmental changes caused by man (Margonari de Souza. 2004). Cutaneous leishmaniasis is one of the most common forms of leishmaniasis disease in Iran. This disease does not have a high rate of mortality, but it causes large skin lesions that can remain over a year and leave scars after recovery, as well as other health problems (MHME 2007). The Sistan-Baluchistan Province in southeast Iran is considered a focus of cutaneous leishmaniasis (Kassiri et al. 2011, Rassi et al. 2004). This disease has been frequently reported from Chabahar and Mirjaveh Counties (Fazeli et al. 2009). So, the objective of this study was to determine the monthly activity of sand flies, both indoors and outdoors, three regions of Sistan-Baluchistan Province with different climatic conditions, namely a plains, coastal, and mountainous region.

## Materials and Methods

This study was conducted in a plains, coastal, and mountainous region in Iranshahr and Chabahar Counties in 1997. Iranshahr is 41,730 km^2^ in size, with six districts, two cities, 21 rural districts, and 181 villages. Chabahar is 17155 km^2^ in size, with five districts, three cities, 11 rural districts, and 591 villages. Three houses were selected in one village per region, and sampling was performed every 20 days. Thirty sticky traps were installed indoors and 30 outdoors. Traps were installed after sunset and were collected before sunrise. Sand flies were removed from sticky traps with an insect needle, rinsed in acetone, and then conserved in 70% ethanol. All specimens were mounted in permanent microscopy slides using Puri's medium. For analyzing the findings, the statistical data was applied based on the abundances and percentages of the observation related to the captured sand flies.

## Results

In total, 8,558 and 1,596 sand fly specimens were collected and identified from outdoors and indoors, respectively. Eight species of *Phlebotomus* and 10 species of *Sergentomyia* from outdoors and nine species of *Phlebotomus* and 10 species of *Sergentomyia* from indoors were identified. Details about the numbers, abundance percentages, and monthly activity of all collected species are given in Tables 1–4.

### Bampoor village

A total of 787 sand flies comprising six species of *Phlebotomus* and six species of *Sergentomyia* were captured indoors in the village of Bampoor (plains region). *Phlebotomus papatasi* (Scopoli) (Diptera: Psychodidae) was the predominant species found indoors in the region (Table 1) and was active during the whole study period (Table 3). The species was most abundant in early May, when 93 specimens were collected, and then the number of adults captured decreased until late July, when the lowest abundance (13 specimens) was collected. The population then gradually increased from late July to October. *Sergentomyia clydei* (Sinton) was the second most predominant species collected indoors in the region. The highest abundance of this species was 38 specimens, in early October, and the lowest abundance was seven specimens, in early June. *Sergentomyia tiberiadis* (Alder, Theodor, and Lourie) and *S. sintoni* Pringle were the third and fourth most predominant, respectively.

A total of 4,768 sand flies comprising six species of *Phlebotomus* and six species of *Sergentomyia* were captured outdoors in Bampoor. *Sergentomyia clydei* was the most abundanct species outdoors in the region (Table 2). The highest abundance of *S. clydei* was found in early October (503 specimens), and the lowest abundance of this species was found in early June (153 specimens). *Phlebotomus papatasi* was collected throughou t the whole study period (Table 4), with the highest abundance (231 specimens) occurring in early October, and the lowest abundance (63 specimens) occurring in early August. Other species found outdoors, *P. bergeroti* Parrot, *S. tiberiadis*, and *S. sintoni*, were also found throughout the whole study period.

### Angorie village

A total of 358 sand flies comprising seven species of *Phlebotomus* and 10 species of *Sergentomyia* were collected indoors in the village of Angorie (mountainous region). *Sergentomyia clydei* was most abundant in late September (17 specimens) and was least abundant in late June (two specimens). *Phlebotomus papatasi* was most abundant in late September (10 specimens) and was least abundant in late June (no specimens collected).

A total of 1,391 specimens comprising six species of *Phlebotomus* and 10 species of *Sergentomyia* were captured outdoors in Angorie. The dominant species was *S. tiberiadis,* and this species indicated high activiactivity during the whole study period. The population size of this species was diverse, as it showed two activity peaks, one in early August and one in early October. The highest *S. tiberiadis* abundance was 77 specimens, and the lowest was 28 specimens. The 1 owest activity of the species was in late June. *Sergentomyia hodgsoni* Sinton was the second most dominant species outdoors in the region. The highest abundance of this species was 56 specimens, in late September, and the lowest was 28 specimens, in late June. *Phlebotomus sergenti* Parrot, *P. alexandri* (Sinton), and *P. kazeruni* (Theodor and Mesghali) were active during the whole study period. The highest abundance of *P. papatasi* at one collection was seven specimens.

**Table 1.**
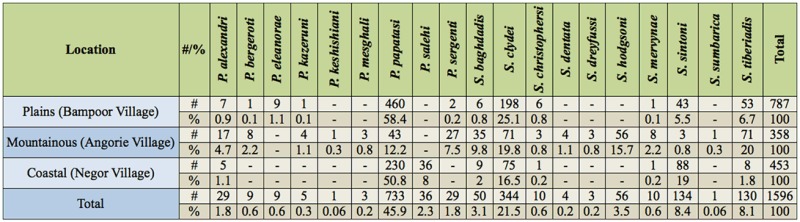
Fauna, number, and percent of sand flies collected indoors in a plains, mountainous, and coastal region of Iranshahr and Chabahar Counties, Sistan-Baluchistan Province, Southeast Iran.

**Table 2.**
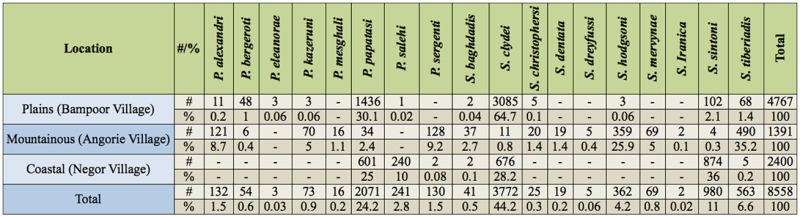
Fauna, number, and percent of sand flies collected outdoors in a plains, mountainous, and coastal region in Iranshahr and Chabahar Counties, Sistan-Baluchistan Province, Southeast Iran.

**Table 3.**
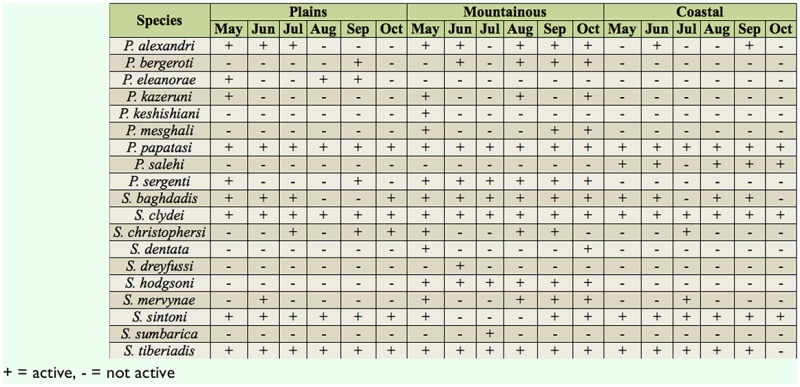
Monthly activity of sand flies collected indoors in a plains, mountainous, and coastal region in Iranshahr and Chabahar Counties, Sistan-Baluchistan Province, Southeast Iran.

**Table 4.**
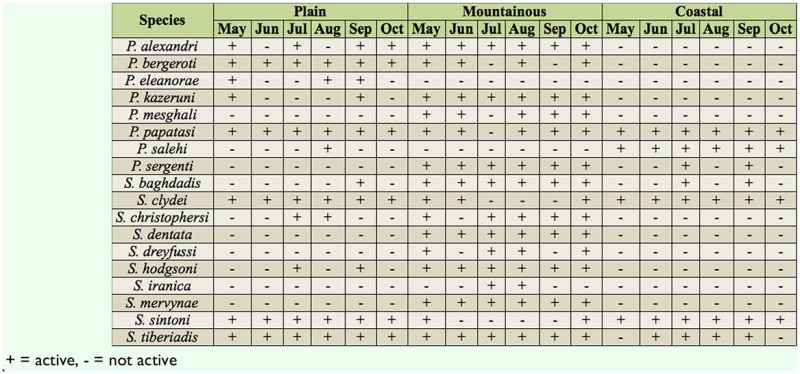
Monthly activity of sand flies collected outdoors in a plains, mountainous, and coastal region in Iranshahr and Chabahar Counties, Sistan-Baluchistan Province, Southeast Iran.

### Negor village

A total of 453 sand flies comprising three species of *Phlebotomus* and six species of *Sergentomyia* were collected indoors in the village of Negor (coastal region). The predominant species captured indoors was *P. papatasi*, being most abundant (42 specimens) in May and least abundant (13 specimens) in late June. *Phlebotomus salehi* Mesghali showed the highest activity (nine specimens) in October. *Sergentomyia sintoni* and *S. clydei* were collected throughout the whole study period.

A total of 2,400 sand flies comprising three species of *Phlebotomus* and four species of *Sergentomyia* were captured outdoors in Negor. *Sergentomyia sintoni* was the most predominant species outdoors in the region. It was most abundant in October (129 specimens) and least abundant in August (54 specimens). *Phlebotomus papatasi* was most abundant in October (93 specimens) and least abundant in late June (26 specimens).

## Discussion

In this entomological survey, a total of 10,157 adult sand flies belonging to 19 species (nine species of *Phlebotomus* and 10 species from *Sergentomyia)* were collected from three regions of Iran, 5,555 individuals being collected from the plains region, 1,749 from the mountainous region, and 2,853 from the coastal region. Although the most specimens were collected in the plains region, the most species were collected in the mountainous region (19 species indoors, and 18 species outdoors). The high abundance and activity of different species indicates the high species richness of sand flies in this region, which is probably due to favorable climatic conditions for the flies. *Sergentomyia clydei* and *S. sintoni,* the most prevalent species found outdoors in the plains and coastal regions, respectively, and *P. papatasi,* the predominant species found indoors in both regions, showed two peaks of activity, in early May and early October.

In this study, *P. paptasi,* a main vector of zoonotic cutaneous leishmaniasis in Iran (Abdoli et al. 2007), was active and collected in all of the sampling period, and showed two peaks of activity indoors and outdoors in the plains and coastal regions; the first peak was in May-June, and the second peak was in August-October. Since the activity of this species peaked at the same time, both indoors and outdoors, in the plains and coastal regions, control methods should be focused on these periods. *Sergentomyia clydei* and *S. tiberiadis* were the predominant species in the mountainous region and are not important in disease transmission. *Phlebotomus papatasi* and *P. salehi* are zoonotic cutaneous leishmaniasis vectors in Sistan-Baluchistan Province (Seyedi-Rashti and Nadim 1984; Kassiri et al. 2000; Kassiri et al. 2012).

Because the climatic conditions vary in different geographical regions of Iran, the peak activity of a species, such as *P. papatasi*, is expected to vary in different regions. Several studies have confirmed this conclusion. Yaghoobi-Ershadi et al. (2010) reported *P. papatasi* as the predominant species in Rafsanjan County, Southeastern Iran, with its two peaks of activity occurring in early June and early August. The activity peak of *S. clydei*, the second dominant species reported in the county, occurred in mid-August. *Phlebotomus papatasi*, the predominant sand fly species of zoonotic cutaneous leishmaniasis importance in Dameghan County, Semnan Province, had one activity peak in late May and one in early September (Mohamadi Azni et al. 2009). The activity peak of this species in Boosheher County, Booshehr Province, was reported in late May (Forozani et al. 2011). *Phelobotomus* sand flies had two peaks of activity, one in late June and the other in late August, in Bam County, Kerman Province (Aghasi et al. 2003). Azizi et al. (2011) collected three species of *Phlebotomus* and five species of *Sergentomiya* from Jask County, Hormozgan Province. The sand flies in this region started to appear in March and disappeared in December, with peaks of activity in May-June and October-November.

In our study, *P. sergenti* and *P. alexandri,* a proven vectors of cutaneous leishmaniasis and a probable vector of Kala-Azar, respectively, were collected mostly in mountainous regions, especially outdoors, in all of the study period. They were collected in small numbers or not collected in the plains and coastal regions. *Phlebotomus alexandri* was collected from May to October in Lordegan County, with peaks of activity in July and August. The peak of activity of *P. major,* a proven vector of Kala-Aza in southern Iran, occurred in October and November, and *P. papatasi* was collected only in June and July (Zahraei-Ramazani et al. 1996).

## Conclusion

Determining the time of peak activity is important for effective surveillance and control of sand flies because measures for prevention and control of the disease vectors should be performed just before the peak of the sand fly activity.
